# Case report: AngioJet thrombectomy with extracorporeal membrane oxygenation support for acute massive pulmonary embolism in a severe multiple trauma patient

**DOI:** 10.3389/fmed.2022.989613

**Published:** 2022-10-14

**Authors:** Lun Tian, Libin Zhang, Naiding Zhang, Xin Xu, Yongshan Xu, Zhenjie Liu, Man Huang

**Affiliations:** ^1^Department of Vascular Surgery, The Second Affiliated Hospital of Zhejiang University School of Medicine, Hangzhou, China; ^2^Department of Intensive Care Unit, The Second Affiliated Hospital of Zhejiang University School of Medicine, Hangzhou, China

**Keywords:** acute pulmonary embolism, AngioJet thrombectomy system, catheter-assisted embolectomy, fragmentation techniques, ECMO

## Abstract

Acute massive pulmonary embolism (PE) is one of the main leading causes of high cardiovascular mortality, and the prognosis strongly varies, depending on the severity of pulmonary arterial obstruction and its impact on the RV function. Alternative therapy approaches comprise systemic thrombolysis, catheter-directed thrombolysis, catheter embolectomy, catheter-assisted fragmentation techniques, and surgical thrombectomy. The following case study explores a 72-year-old man with severe multiple trauma who suffered from a sudden massive pulmonary embolism and presented with an unstable hemodynamic status. Extracorporeal membrane oxygenation (ECMO) has amply proven its efficacy in supplying cardiopulmonary assistance for this patient shocked by a massive PE with contraindication for thrombolysis. AngioJet catheter embolectomy and ECMO were performed, which finally cleared the massive pulmonary embolism away and improved the patient's hemodynamic status. The use of ECMO was continued during the weaning program, on the fifth day after ECMO decannulation, the patient was extubated and transferred to a local hospital for further recuperation. This case highlights that the AngioJet thrombectomy with the combination use of ECMO may be a potential choice of treatment for unstable PE patients.

## Introduction

Acute pulmonary embolism (PE) is a partial or complete occlusion of the pulmonary arteries, with hemodynamic consequences determined by the size and location of the embolus, preexisting cardiopulmonary disease, and the severity of ventilation and oxygenation compromise. Annual PE incidence rates range from 39 to 115 per 100,000 population, steadily increasing over the past decades, notwithstanding, both PE-related in-hospital death rates and age-standardized mortality from PE have been dwindling or stagnating in recent years (1). The physical pulmonary arterial obstruction raises pulmonary vascular resistance and right ventricular (RV) afterload, ultimately causing RV failure with a subsequent life-threatening reduction in coronary perfusion and cardiac output. Clinical presentation can manifest as asymptomatic to catastrophic and symptoms are determined by the embolic burden as well as the severity of any accompanying cardiopulmonary disease (2). The standard treatment is systemic anticoagulation, but in the case of high or intermediate-risk PE, treatment can be escalated. Systemic thrombolysis, surgical thrombectomy, and catheter-directed interventions (CDIs) drive experts' attention, and are ready for primetime. Patient selection criteria, hemodynamic conditions, and clinical outcomes are considered to resolve the indications and benefits of invasive interventions. As new technology evolves, up to now, CDIs vary and can be utilized with or without thrombolysis. More specifically, for patients who have absolute contraindications to thrombolysis, this approach with no lytic agents, consisting of thrombus fragmentation and/or aspiration techniques, is properly fitting (3, 4). For patients with fatal bleeding risks such as recent surgical procedures, intracranial hemorrhage, and other contraindications to thrombolysis, anticoagulation, or surgical thrombectomy; the treatment options are of controversial complexity. The AngioJet rheolytic thrombectomy (ART) system provides pharmacomechanical thrombolysis and has been previously attempted for PE, with promising clinical outcomes despite several adverse events reported (5–8). Nowadays, transportable extracorporeal membrane oxygenation (ECMO) assistance systems with percutaneous femoral cannulation may become effective in extreme circumstances, maintaining circulation and oxygenation. It retrieves the sharply failing right heart as well, implementing adequate hemodynamic and respiratory support for patients who experience massive PE until the initial pulmonary conditions are recovered. It has been increasingly used for patients with unstable PE who are not responding to other therapeutic approaches, or as catheter-based or surgical embolectomy media (9). Throughout this case study, we encountered challenges with the catastrophic pulmonary and cardiogenic collapse in a multiple trauma patient, who underwent the AngioJet thrombectomy stabilized by an ECMO system and successfully survived.

## Case description

**Figure 1:**
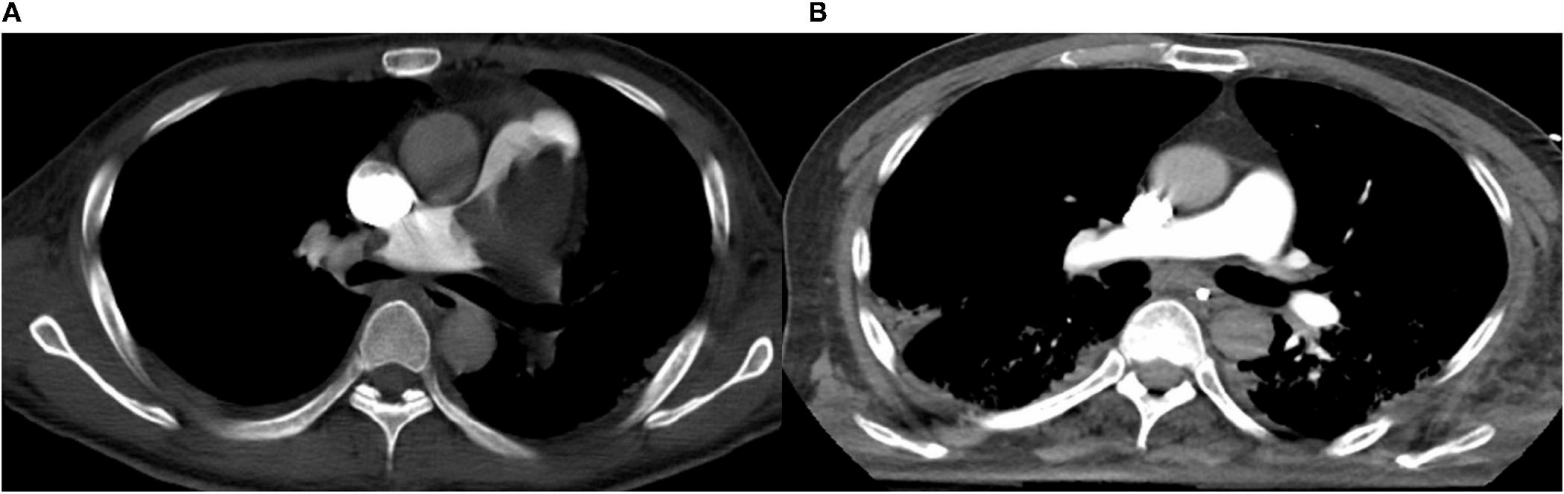
**(A)** Computed tomography pulmonary angiography (CTPA) demonstrates pulmonary embolism (PE). **(B)** Computed tomography pulmonary angiography (CTPA) demonstrates that residual thrombus markedly improved.

**Figure 2:**
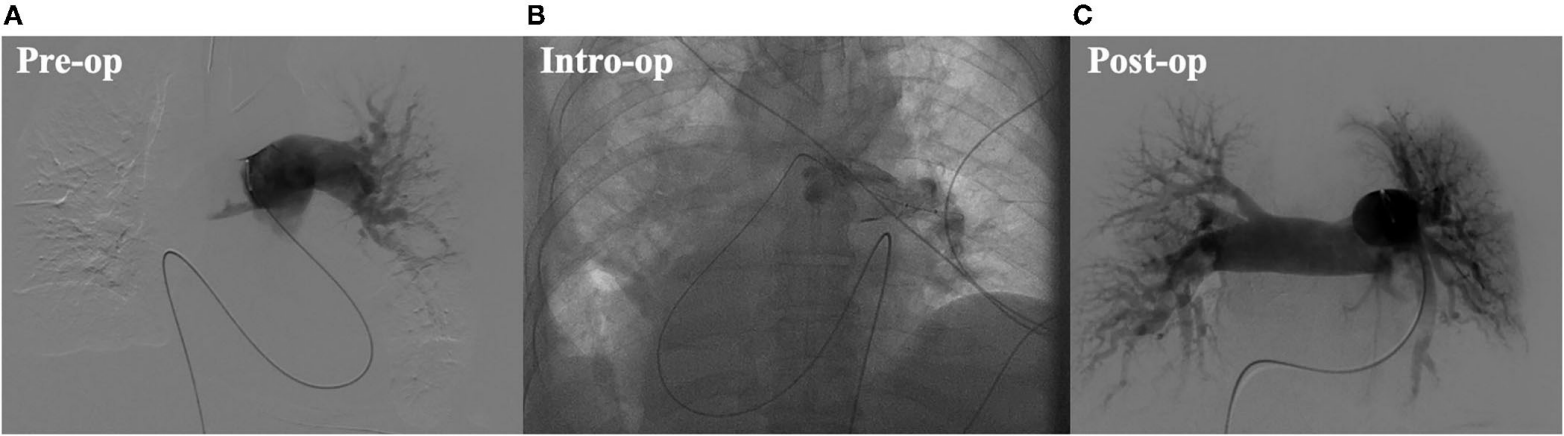
**(A)** Preoperative digital substraction angiography (DSA) image. **(B)** Intro-operative digital substraction angiography (DSA) image. **(C)** Postoperative digital substraction angiography (DSA) image.

In September 2021, a 72-year-old male falling from a height of 3 m with multiple trauma (right temporal subdural hematoma, subarachnoid hemorrhage, right frontal brain contusion, left temporal bone fracture, left clavicle and left 1–11 ribs, right 1st rib fracture, and hemopneumothorax) and a history of long-term smoking. The patient became progressively short of breath during the local hospitalization, developed wheezing with dyspnea, his oxygenation decreased and consciousness suddenly changed, consequently, he underwent tracheal intubation and ventilator-assisted ventilation. While transferring to our emergency room for further life-saving treatment, his condition aggravated with unstable vital signs. His subsequent examination revealed a large bilateral PE, especially the bifurcation of the main pulmonary artery, the posterior basal segment of the dorsal segment of the right inferior pulmonary artery, and the posterior and external basal segment of the left upper pulmonary artery by computerized tomography pulmonary arterial angiography (CTPA) (Figure 1A). Pervasive deep thrombosis was also visible in the bilateral posterior tibial and calf intermuscular veins by ultrasound Doppler. Electrocardiography (ECG) showed sinus rhythm and atrial premature beat. The arterial blood gas analysis indicated that blood PH of 7.14, blood base excess of −8.50 mmol/L, lactic acid of 20.00 mmol/L, oxygen tension of 379.70 mmHg, carbon dioxide tension of 59.40 mmHg, and potassium of 5.80 mmol/L. Echocardiography showed right-sided heart enlargement and pulmonary hypertension (Table 1). Therewith, the patient experienced severe cardiopulmonary decompensation and sudden cardiac arrest. Following academic discussion, the multidisciplinary team of specialists determined that bleeding risk and other comprehensive elements contributed to the patient's contraindication for systemic thrombolysis or surgical embolectomy. They reached a consensus that AngioJet catheter thrombectomy of the massive PE integrated with veno-arterial extracorporeal membrane oxygenation (VA-ECMO) supports might be a better option. Following the transition of VA-ECMO at 2.9~3.1 L per minute *via* left femoral and right internal jugular vein to left femoral artery, the patient's hemodynamic indicators ameliorated exacerbation. An AngioJet thrombectomy system—Solent^®^ Omni was used to perform mechanical aspiration thrombectomy on the patient's right pulmonary thrombus. The pulmonary angiograms revealed discontinuities in the trunk of the left main pulmonary artery, with a marginal change in left lower lobe perfusion. The embolus catheter was inserted in the right pulmonary artery and continued to suction for 100 s, then a repeated pulmonary angiogram demonstrated a substantially improved blood flow to the right lung (Figures 2A–C). At this time, the intraoperative echocardiogram demonstrated no dyskinesia of the myocardial wall with otherwise normal function and an ejection fraction of ~65%. The patient was transferred back to the intensive care ward, and the nurse constantly monitored the ECMO settings and circuit. Upon closer inspection, there were no severe adverse events, nor were there any complications related to the procedure, the patient hemodynamic parameters were stable, and hypoxia was resolute. Furthermore, the reversal of RV failure and pulmonary artery hypertension was assessed using echocardiography after 48 h. Following this initial week, CTPA was repeated to evaluate the residual thrombus, which was markedly improved (Figure 1B). The VA-ECMO was weaned on the fifth day and the patient was transferred to the local hospital for further recuperation. The echocardiography was performed at 4-weeks postoperatively which proved the pulmonary artery hypertension and the reversal of RV failure were in good condition.

**Table 1:** The echocardiographic findings and laboratory results before and after the AngioJet procedure.

	**Echocardiographic findings**				**Laboratory results**		
	**PA pressure**	**RV size (ventricular diameter)**	**Function TAPSE**	**RV/LV ratio**	**Pro-BNP**	**Troponin**	**Hemoglobin**
Before AngioJet	56 mmHg	4.5 cm	2.30 cm	1.4	417 pg/ml	0.016 ng/ml	138 g/L
After AngioJet	44 mmHg	4.9 cm	2.10 cm	1.2	756 pg/ml	0.067 ng/ml	121 g/L

## Discussion

Massive pulmonary embolism constantly foreshows a fatal outcome, with a mortality rate ranging from 30 to 50% (9). Hemodynamic status is considered a crucial short-term predictive factor for patients with acute PE, according to Antithrombotic Therapy for VTE Disease CHEST Guideline and Expert Panel Report 2016, scholars are of the opinion that for patients at high risk who require immediate reperfusion therapies, mechanical methods can be used alone when thrombus removal is indicated but thrombolytic therapy is contraindicated due to a high-risk of bleeding (10). Thus, emergent revascularization and systemic thrombolysis might be the first alternative for patients with unstable hemodynamics. Multiple technical options targeting rapid clot debulking for unstable patients have been reported in various combinations, particularly when lytics are contraindicated. The AngioJet System creates a local low-pressure zone to entrain, fragment, and aspirate thrombi *via* Bernoulli's principle of fluid dynamics. The largest and latest 7-year single-center study on AngioJet rheolytic thrombectomy in patients with PE revealed that for patients with PE who have various comorbidities and a proclivity to bleed, ART may be considered preferable, resulting in significant improvements in obstructive burden and hemodynamics (8). ART mentioned in most cases or articles (8, 11), instead of using the pulse spray technique and t-PA assisted in increasing the surface area of thrombus to enhance the effectiveness of lytic agents, we chose thrombus fragmentation and aspiration techniques with no lytic agents for this patient who had absolute contraindications to thrombolysis. In addition, some patients who underwent catheter rheolytic thrombectomy died from cardiogenic shock. The patient has to be hemodynamically stable for an extended period of time, allowing for a significant reduction of the thrombus load so as to the rheolytic thrombectomy to be efficacious. Therefore, for patients with PE in unstable status, ECMO can quickly attenuate hemodynamic collapse and facilitate treatment over several days better than cardiopulmonary bypass and surgical embolectomy.

Patients with massive PE present in extremes and who are too unstable to successfully undergo CDI, with the help of ECMO, can be resuscitated and supported until thrombus removal occurs by using other catheter-directed interventions. With VA-ECMO, venous blood is drained into the inferior vena cava *via* a cannula inserted through the femoral vein, and oxygenated blood is reinfused *via* a cannula inserted through the femoral artery into the thoracic descending aorta. Recently, VA-ECMO has been performed successfully to treat patients suffering from massive PE, some of whom have reported excellent short-term outcomes (12). The prognosis of acute PE strongly varies, many times, physicians will face dilemmas as to what approach will best balance the benefits of surgical invasions against potential risks of cardiopulmonary failure or death. For the treatment of massive PE in this patient with unstable hemodynamic status, considering bleeding risk and contraindications for thrombolysis or surgical embolectomy, catheter embolectomy under the protection of ECMO should be considered as a first-line treatment in the appropriate clinical setting, as a way to provide the benefits and minimize adverse events. As the renovation of novel interventional devices and technology advances, we should underscore the requirement for an integrative multidisciplinary approach flexible enough to evaluate and utilize; the supplement of ECMO combined with the AngioJet catheter thrombectomy may be a good choice of treatment in massive PE. Despite the publication of several case reports, further research, specifically randomized controlled trials, are necessary to confirm its efficacy and safety to become sufficient robust evidence.

## Data availability statement

The raw data supporting the conclusions of this article will be made available by the authors, without undue reservation.

## Ethics statement

The studies involving human participants were reviewed and approved by the Ethics Committee of the Second Affiliated Hospital of Zhejiang University, School of Medicine. The patients/participants provided their written informed consent to participate in this study.

## Author contributions

LT collected the patient's information and wrote the manuscript. ZL and LZ performed the surgical procedures. LZ, NZ, XX, and YX underwent data curation and validation. ZL and MH reviewed and edited the original draft. The article was contributed by all of the authors and the final manuscript was approved by all of them.

## Funding

This work was supported by the National Natural Science Foundation of China (81670433 and 81970398), the Project of Zhejiang Medical Young Talents (2017), Zhejiang Medical and Health Science and Technology Project (2020RC014), the Natural Science Foundation of Zhejiang Province (Q20H020059), Science Fund for Distinguished Young Scholars of Zhejiang Province (LR22H020002), and Natural Science Foundation of Zhejiang Province (LQ20H020008).

## Conflict of interest

The authors declare that the research was conducted in the absence of any commercial or financial relationships that could be construed as a potential conflict of interest.

## Publisher's note

All claims expressed in this article are solely those of the authors and do not necessarily represent those of their affiliated organizations, or those of the publisher, the editors and the reviewers. Any product that may be evaluated in this article, or claim that may be made by its manufacturer, is not guaranteed or endorsed by the publisher.
